# Investigating autoimmunity in the etiology of premature ovarian insufficiency in a mouse model

**DOI:** 10.1016/j.jri.2026.104875

**Published:** 2026-06

**Authors:** Nilay Kuscu, Ruth Appeltant, Sergej Petrovic, Suzannah A. Williams

**Affiliations:** a Nuffield Department of Women's & Reproductive Health, University of Oxford, Oxford OX3 9DU, United Kingdom; b Gamete Research Centre, Veterinary Physiology and Biochemistry, Department of Veterinary Sciences, University of Antwerp, Wilrijk, Belgium

**Keywords:** Premature ovarian insufficiency, Autoimmunity, Follicle development, Ovary

## Abstract

Premature ovarian insufficiency (POI) is a major cause of infertility, yet its underlying mechanisms remain poorly understood. The double-mutant (DM) mouse, featuring oocyte-specific deletion of *mannosyl (α-1,3-)-glycoprotein β-1,2-N-acetylglucosaminyltransferase* (*Mgat1)* and *core 1 β-1,3-galactosyltransferase (C1galt1)*, develops early-onset POI and offers a valuable system to investigate causes. This study explored whether autoimmunity leads to POI in DM mice. We transplanted control and DM neonatal ovaries into immunocompromised hosts. Three months post-transplantation, follicular development was assessed. While both DM and control ovarian grafts contained follicles, only control ovaries showed healthy progression through to the antral stage. In contrast, DM ovaries exhibited a marked developmental block at the primary stage. In DM follicles, oocytes were smaller, and granulosa cell (GC) number and area were reduced at all follicular stages present. There was a strong correlation between oocyte growth and GC proliferation in controls, but was weaker in DM, suggesting disrupted oocyte–somatic cell communication. Analyses showed strong anti-Müllerian hormone (AMH) in Control GCs, whilst DM levels were less than half of the control, indicating compromised GC function in DM ovaries. Forkhead box L2 (FOXL2) was also reduced in DM follicles compared to control GCs. The persistence of follicular defects, including reduced AMH and FOXL2 levels, in DM ovaries even in severe combined immunodeficient mice reveals that POI is not due to autoimmunity and the phenotype results from intrinsic defects in follicular development caused by the oocyte-specific mutation. These findings underscore the role of oocyte-GC interactions in follicle development and contribute to our understanding of POI pathogenesis.

## Highlights

• POI in the DM mouse model is not due to autoimmunity.
• Follicle development in DM ovaries is blocked at the primary stage in severe combined immunodeficient (SCID) mice.
• Granulosa cell development is impaired in DM ovaries developing in SCID mice.

## 1 Introduction

Ovarian dysfunction is a key contributor to the pathogenesis of premature ovarian insufficiency (POI). The condition is primarily attributed to two etiological mechanisms: follicular depletion and follicular dysfunction. Follicular depletion results in ovaries that are devoid of follicles (afollicular), whereas follicular dysfunction is characterized by ovaries containing a heterogeneous array of developing follicles (follicular) (Nelson et al., 1994, Meskhi and Seif, 2006, Nelson, 2009, Hubayter et al., 2010, Suzuki et al., 2015, Rudnicka et al., 2018). Clinically, POI is defined as reduced or absent ovarian function in women under 40 years of age. It affects 1–3% of this population and it typically presents as continuous amenorrhea for at least four months (Rudnicka et al., 2018).

POI is largely idiopathic, accounting for up to 70% of cases (Chapman et al., 2015), and the mechanisms underlying ovarian dysfunction still poorly understood (Shuster et al., 2010). However, autoimmunity is implicated in approximately 4–30% of cases (Sharif et al., 2019).

Autoimmune diseases are complex and can increase the risk of other autoimmune conditions; for example, women with Hashimoto’s disease are 2.4 times more likely to develop POI (Hsieh, Ho, 2021). Studies in both humans and animal models provide evidence of immune involvement in POI, including genetic mutations affecting T-cell regulation, which are associated with POI in 45–60% of women with such mutations (Vujovic, 2009). The involvement of autoimmunity in POI is supported by studies demonstrating that immunizing mice with zona pellucida protein 3 (ZP3) glycoprotein or altering thymus function can induce POI (Tung et al., 1997, Setiady et al., 2003). Additionally, immune-related genes such as immunoglobulin heavy constant mu (IGHMU) and lysozyme (LYZ) are differentially expressed in women with POI, reinforcing the potential role of autoimmunity in its pathogenesis (Zhang et al., 2023).

Mouse models have proven invaluable for studying POI, with diverse approaches employed to induce ovarian dysfunction, including psychological stress, lipopolysaccharide injections, and chemotherapy agents (Wang et al., 2015, Lv et al., 2021, Buigues et al., 2019). Among these, the double-mutant (DM) model stands out as a robust tool for exploring the etiology of POI (Williams and Stanley, 2011), including the role of autoimmunity. This model features oocyte-specific deletions of the *mannosyl (α-1,3-)-glycoprotein β-1,2-N-acetylglucosaminyltransferase (Mgat1)* and *core 1 β-1,3-galactosyltransferase* (*C1galt1)* genes, resulting in follicular POI that progresses to an afollicular state, mirroring the human condition. The ZP3 gene, used as the drive for the conditional knockout, is oocyte-specific and vital for oocyte development (Wassarman, Litscher, 2021). DM mice exhibit a clear timeline of disease progression; ovaries at 3 weeks are grossly normal, containing many follicles at different stages of development, transitioning from subfertility at 6 weeks to infertility by 9 weeks and developing follicular POI by 3 months of age (Williams and Stanley, 2011). Diagnostic criteria, including the absence of developing follicles, ovarian dysfunction, and altered hormone profiles, validated the DM model as a reliable representation of POI (Williams and Stanley, 2011). Over time, follicular POI in these mice progresses to an afollicular state analogous to the trajectory observed in human cases. The conditional knockout of *Mgat1* and *C1galt1* disrupts glycosylation processes which are critical for follicle development (Ju et al., 2002, Shi et al., 2004) because glycans play structural roles and mediate cell communication within the follicle (Lo et al., 2019). Dual knockout of oocyte N- and O-glycans results in impaired ovulation, follicular development, with downstream dysfunctional consequences for embryo development and implantation (Williams and Stanley, 2011, Grasa et al., 2012, Li et al., 2020). Transcriptomic analyses of DM ovaries revealed sharp changes in gene expression from 6 to 9 weeks of age, particularly in pathways related to cell communication and extracellular matrix, implicating disrupted oocyte–somatic cell signaling as a driver of the phenotype (Williams and Stanley, 2009, Kaune et al., 2022). Histological studies further demonstrated that DM ovaries contain more primary (3a) follicles accompanied by fewer developing follicles, indicating a block in follicle progression. Alterations in the follicular basal lamina and extracellular matrix gene expression with aging are the key pathological features in DM mice (Williams and Stanley, 2011, Grasa et al., 2012, Grasa et al., 2016, Kaune et al., 2022). Transplanting 9-week-old ovarian tissue to an immunocompatible control sibling, i.e. a normal environment, did not restore normal follicle development (Sheikh et al., 2023). Functional experiments using reaggregated ovaries showed that replacing DM somatic cells restored follicle development, highlighting that oocyte–somatic cell communication is critical for normal follicle development (Sheikh et al., 2023). Given POI can be associated with autoimmune oophoritis, immune-mediated follicle arrest, and accelerated follicle depletion (Nelson, 2009, Hoek et al., 1997, Grossmann et al., 2020), it remains unclear whether the ovarian abnormalities in DM arise solely from intrinsic oocyte defects or are influenced by autoimmune mechanisms. Thus, the DM model offers valuable insights into the underlying mechanisms driving the pathogenesis of POI, establishing it as a valuable tool for advancing our understanding of POI etiology.

To study ovarian function in mouse models that undergo prepubertal lethality, or to assess ovarian tissues from other species, whole ovaries or ovarian fragments can be transplanted into immunodeficient mice. This approach provides a permissive host environment that supports graft vascularisation and follicle development (Ackert et al., 2001, Commin et al., 2012, Wei et al., 2024). Accordingly, this transplantation model was used in the present study.

Therefore, this study aims to investigate the potential contribution of autoimmunity to the onset of POI in the DM mouse model by transplanting neonatal DM ovaries to an immune-deficient environment for 3 months. We analysed follicle development by detecting AMH and FOXL2. AMH is produced by granulosa cells of growing follicles and serves as a marker of granulosa cell functionality, with the highest expression in preantral and small antral follicles (Durlinger et al., 2002). In parallel, FOXL2 is a key transcription factor that marks early granulosa cell development and differentiation. FOXL2 is essential for the activation of primordial follicles and for supporting subsequent stages of follicle growth (Schmidt et al., 2004), making both AMH and FOXL2 informative indicators of granulosa cell health and follicle development in our model. The hypothesis we tested was that autoimmune mechanisms play a contributing role in the development of POI in this model.

## 2 Materials and Methods

### 2.1 Mice

All mouse experiments were conducted with approval from the Local Ethical Review Panel at the University of Oxford and in accordance with the United Kingdom Animals (Scientific Procedures) Act 1986. Mice were housed in individually ventilated cages, provided with food and water ad libitum, and maintained on a 12:12 h light-dark cycle.

Mice used in this experiment were divided into control and experimental DM group. DM females carry floxed *C1galt1* and *Mgat1* alleles along with the ZP3Cre transgene, while control females carry the floxed alleles, but lack the ZP3Cre transgene, with the floxed alleles functioning as wildtype genes (Shi et al., 2004, Williams et al., 2007). The ZP3 transgene does not affect fertility (Shi et al., 2004, Williams et al., 2007). For ovarian transplant experiments, newborn (postnatal day 0) DM and control females served as donors, and ovarian tissue was transplanted into 15–19-week-old C.B-17/IcrHan®Hsd-Prkdcscid female mice (Envigo) recipients, which lack functional T and B lymphocytes. These mice harbor a mutation in the DNA-dependent protein kinase catalytic subunit (Prkdc), resulting in a severe combined immunodeficiency characterized by an absence of mature T and B lymphocytes. Innate immune components, including NK cells, macrophages, and granulocytes, are preserved and remain functional. We therefore interpret this model as one that lacks adaptive, but not innate, immunity. The data presented here is from 6 transplantations.

### 2.2 Genotyping

DM and control mice were genotyped using protocols as described (Grasa et al., 2012, Grasa et al., 2015).

### 2.3 Ovary transplantation under the kidney capsule

Ovaries for transplantation were harvested from D0 mice euthanized by cervical dislocation and placed in sterile L-15 Leibovitz medium (Hyclone, Thermo Scientific, Utah, USA), supplemented with 3 mg/mL of BSA. DM and control ovaries were surgically transplanted under the kidney capsule of immunocompromised mice as previously described (Wei et al., 2024). In brief, anaesthesia was induced with 3% isoflurane and 2 L/min of oxygen and then maintained at 2.5% isoflurane and 1.5 L/min of oxygen. Analgesic agents, Vetergesic (10%, Ceva Animal Health, United Kingdom) and Metacam (10%, Boehringer Ingelheim, Germany), were administered subcutaneously. Left dorsal and lateral areas of mice were shaved and the exposed skin antiseptically cleaned. To transplant the ovaries under the kidney capsule, a small flank incision of the skin and a further small incision of the body wall were made to access the left kidney. The left kidney was exteriorised and a small pocket was made under the kidney capsule using fine forceps into which the neonatal ovary was inserted. The kidney was returned to its normal position, and the body wall and skin closed with interrupted sutures using 6–0 Vicryl sutures (W9575, Ethicon, United States. The host ovaries were left in situ. After surgery, mice were placed in pre-warmed cages. Soft food (normal food soaked in water) in a petri dish and water in a petri dish was provided in addition to the normal food pellets and water bottle. Mice were weighed and monitored weekly and transplanted tissues were recovered after 3 months. All analyses were conducted using tissue derived from n = 3 ovarian grafts per group.

### 2.4 Ovarian histology and assessment

The recovered tissues contained kidney tissue adjacent to the ovarian tissue; these were processed together to be sure to leave the ovarian tissue intact. The recovered tissues were fixed in 5% Form-Acetic (Adeniran et al., 2021) for 24 h, and embedded in paraffin wax. The tissues were serially sectioned (5 μm), with every 10th section stained with hematoxylin and eosin for histological analysis. Sections were deparaffinised in three washes of xylene and rehydrated through decreasing concentrations of ethanol to water. Sections were stained with haematoxylin (Shandon Gill 2, Thermo Fisher Scientific, UK) to visualize nuclei, and excess staining was removed using acid alcohol (1% HCl in 70% ethanol). Sections were then briefly transferred into eosin Y solution (Sigma-Aldrich, USA) to stain cytoplasm and extracellular matrix. Next, sections were dehydrated through increasing concentrations of ethanol and washed twice in xylene. Slides were mounted with coverslips (no. 1.5, 630–1845, VWR International) using DPX (06522, Sigma-Aldrich) and left to dry overnight. Sections were imaged using a DM2500 Leica microscope (Microscope Services Ltd, UK) with a MicroPublisher 5.0 RTV 149 camera (Qimaging, Microscope services Ltd, UK), using software Infinity Analyze (Microsoft Windows, USA).

### 2.5 Counting and Analysis of Follicles

Morphologically healthy follicles with a central oocyte and a visible nucleus were assessed in the stained sections to make sure that during no follicle was double counted. Counting was performed in a blinded manner. Follicles were classified according to the criteria described by Sheikh et al. (2023). Follicles were categorised as primordial, transitional, primary, primary+ , secondary, preantral and antral follicles. Main determinant for the classification was number of complete, compact GC layers. Primordial follicles had only squamous GC, transitional had squamous and cuboidal. Primary, secondary and tertiary had 1, 2 and 3 or more complete layers, respectively. Primary+ follicles represent an intermediate stage between primary and secondary follicles, characterized by having an incomplete second-layer. The only difference was that, because of the nature of experiment some of the groups presented in very low numbers. Therefore, they were classified with a neighbouring group (small number of antral follicles resulted in making a group abbreviated as tertiary, which counted both pre-antral and antral follicles).

Detailed analysis of developing follicles was performed using ImageJ software (National Institutes of Health, USA). Oocyte diameter was calculated as the average distance of a perpendicular measurement across the oocyte passing through the nucleus. The number of GCs were counted in each follicle and the GCs compartment area was obtained by subtracting the oocyte area from the follicle area.

### 2.6 Immunohistochemistry (IHC)

Foxl2 is a transcription factor which is essential for follicle development and GC function (Schmidt et al., 2004). Changes in its expression can reflect disruptions in follicle development, which is a key part of ovarian function. AMH is a regulator of ovarian reserve and primordial follicle activation (Durlinger et al., 2002, Weenen et al., 2004). AMH reflects the size of the ovarian reserve and controls primordial follicle activation, so altered AMH levels indicate changes in follicle pool dynamics and ovarian health. Together, AMH and Foxl2 were used to investigate molecular changes associated with ovarian dysfunction using IHC.

Sections were dewaxed and rehydrated. Heat induced antigen retrieval was performed for 20 min in microwave, using 1x unmasking solution Tris-based (Vector laboratories, UK, H-3301). To block the activity of endogenous peroxidase, slides were exposed to 3% hydrogen peroxide in PBS (Sigma-Aldrich, USA, 5LB5706) for 5 min followed by a PBS washed. For specific detection of AMH, unspecific binding was blocked by incubating slides for 45 min in 5% normal goat serum (Vector laboratories, UK, ZGO702) in PBS. Foxl2 was blocked using 5% rabbit serum in PBS. Primary antibody used for detection of Foxl2 was goat polyclonal (Novus Biologicals, USA, NB100–1277SS) and for AMH mouse monoclonal (Biorad, MCA 2246), in 1:100 and 1:50 dilutions, respectively. Negative controls were not incubated with primary antibody. After overnight incubation at 4°C, samples were washed with PBST 3 times for 3 min and incubated with secondary antibodies. For AMH, goat anti-mouse secondary antibody was used in 1:100 dilution (Vector laboratories, UK, BA9200) and for Foxl2 rabbit anti-goat secondary antibody was used in 1:300 dilution (Vector laboratories, UK, BA-5000) for 1 h at room temperature. Visualisation was enabled by avidin-biotin reaction using ABC kit (Vector laboratories, UK) for 30 min. DAB (Vector laboratories, UK) was used to visualize the reaction and then slides were counterstained with haematoxylin, dehydrated and mounted using DPX Mounting solution. Slides were imaged using microscope DM2500 Leica with a MicroPublisher 5.0 RTV 149, using software Infinity Analyze. Semi-quantitative analysis of AMH and FOXL2 staining in ovarian sections was performed using ImageJ. For each image, the total ovarian tissue area was manually delineated, and regions of positive staining were identified using a consistent thresholding method (Crowe and Yue, 2023). To assess relative staining per cell, GC nuclei within the stained regions were manually counted. The total area of positive staining was then divided by the number of stained GC nuclei to estimate the average AMH per GC or FOXL2 staining per GC nucleus. Analyses were performed on a total of 66 follicles in control ovaries and 100 follicles in DM ovaries, derived from n = 3 ovarian grafts per group.

### 2.7 Statistical analysis

Distribution normality of data (Gaussian) was checked using Shapiro-Wilk test for n < 10, and for n > 10 D’agostino-Pearson test was used. Correlation tests used were dependent on differences in data sets. If data is parametric and normally distributed, Pearson correlation rank test was used, and if it was a non-parametric comparison- Spearman rank correlation was performed. To test differences between two groups with normal distributions, unpaired *t*-test was used, while for non-parametric, Mann-Whitney test was performed. Welsch correction was applied to data sets without normal distribution. Statistical tests were performed in Microsoft Excel and GraphPad Prism. Statistical significance was defined as follows: p < 0.05 (*), p < 0.01 (**), p < 0.001 (***), and p < 0.0001 (****). Bar graphs represent mean values and standard error of mean (SEM).

## 3 Results

### 3.1 Analysis of follicular development

**Fig. 1:**
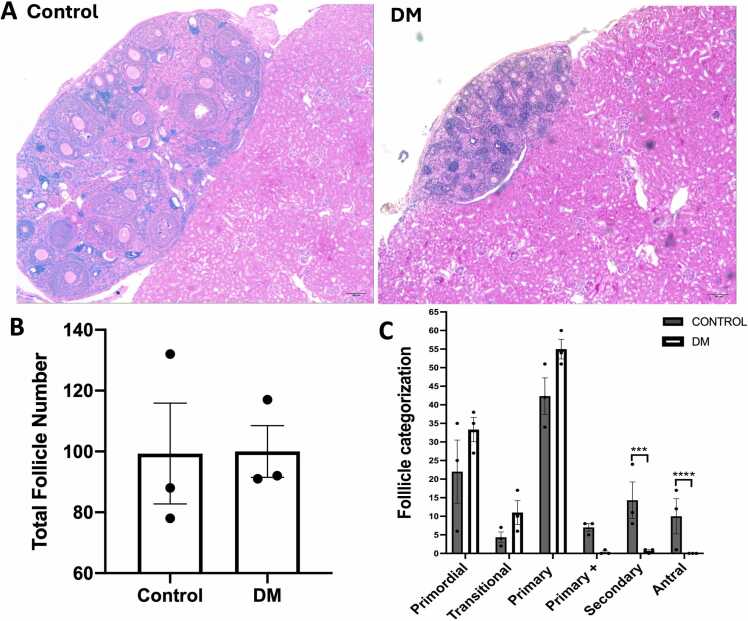
Follicle development and comparison of follicle numbers and stage in ovaries from control and DM mice. A. Representative H&E images show ovarian morphology and follicle development in ovarian tissue graft from control and DM mice. Magnification: 5X. B: Quantification of total follicle number per ovarian graft. Follicles were counted in three independent grafts per group (n = 3) by analysing every 10th serial section throughout each graft. Data are shown as mean ± SEM. C: Number of follicles at each stage of development in the control and DM ovarian tissue graft. Results are presented as mean ± SEM. ***p < 0.001, ****p < 0.0001.

DM and control ovaries were assessed through morphological evaluation using H&E staining. Early follicle development was observed in both groups, confirming the functional viability of the transplanted tissue. While the control group exhibited numerous large, growing follicles containing centrally located, round oocytes, the DM transplants contained only smaller follicles, with visibly smaller oocytes (Fig. 1A).

Follicular development in DM and control ovaries was assessed by counting the follicles (Fig. 1B). Number of total follicles counted in each ovarian graft by analysis of every 10th section (n = 3). The control group had 298 follicles, while the DM group had 301 follicles. The mean follicle count for each group was calculated and compared and there is no difference between the groups (both groups followed a normal Gaussian distribution, p = 0.4164).

To further evaluate differences in follicular development, the stages of follicle development were analysed between the groups (Fig. 1C). Control ovaries contained primordial follicles as well as healthy transitional, primary, primary+ , secondary and antral follicles as would be expected in a normal ovary that has been transplanted to an immunocompromised host. However, in the DM ovary transplants, follicles remained at the primary stage, with a diminished presence of later stages. In the control group, 16–46% of follicles had progressed beyond the primary stage (primary+, secondary, and antral follicles). In contrast, only 0–1.7% of follicles in the DM group reached the same stage. The DM group exhibited a significant reduction in the number of secondary and antral follicles compared to the control group (***p < 0.001, ****p < 0.0001), highlighting impaired follicular progression in DM ovaries.

Overall, these results demonstrate that DM mice ovaries experience a follicular developmental block preventing follicles from achieving full maturation when supported in an immunocompromised environment. This provides evidence of impaired follicle development in the DM mice, which contributes to the observed ovarian dysfunction.

### 3.2 Analysis of oocyte and GC and their correlation

**Fig. 2:**
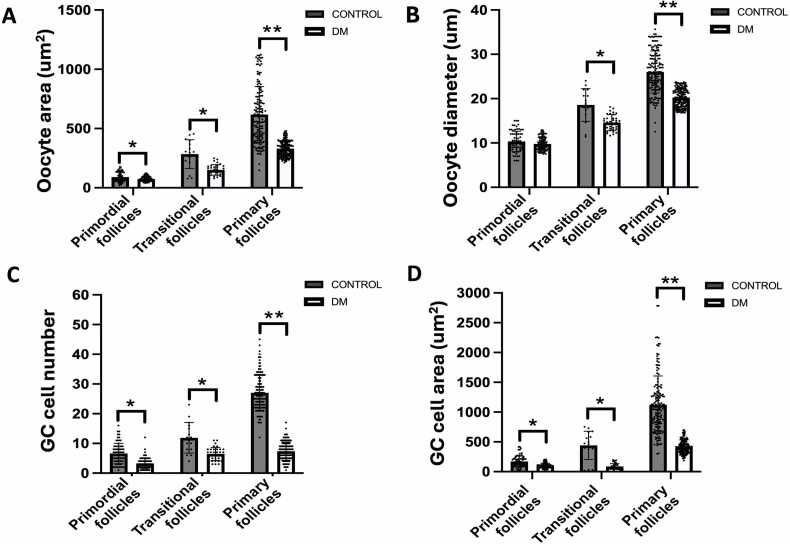
Comparison of oocyte size using both diameter (A) and area (B) and comparison of Granulosa cells (GC) using both number (C) and area (D) in primordial, transitional and primary follicles between control and DM ovaries. Follicles were derived from n = 3 ovarian grafts per group. Results are presented as mean ± SEM. *p < 0.05, **p < 0.01.

To directly compare follicle development in both control and DM ovarian tissue, oocyte diameter and oocyte area were measured in primordial (n = 66 in control and n = 100 in DM group), transitional (n = 13 in control and n = 33 in DM group) and primary follicles (n = 148 in control and n = 166 in DM group) (Figs. 2A and 2B). Both oocyte diameter and oocyte area were significantly decreased in the DM group compared to the control group in both transitional and primary stage follicles (*p < 0.05 and **p < 0.01).

Furthermore, both number of GCs and measuring their subsequent area were carried out and compared between the groups in primordial (n = 66 in control and n = 100 in DM group), transitional (n = 13 in control and n = 33 in DM group) and primary follicles (n = 148 in control and n = 166 in DM group) (Figs. 2C and 2D). Both GC number and the cell area were significantly decreased in the DM group compared to the control group across the follicular stages.

**Fig. 3:**
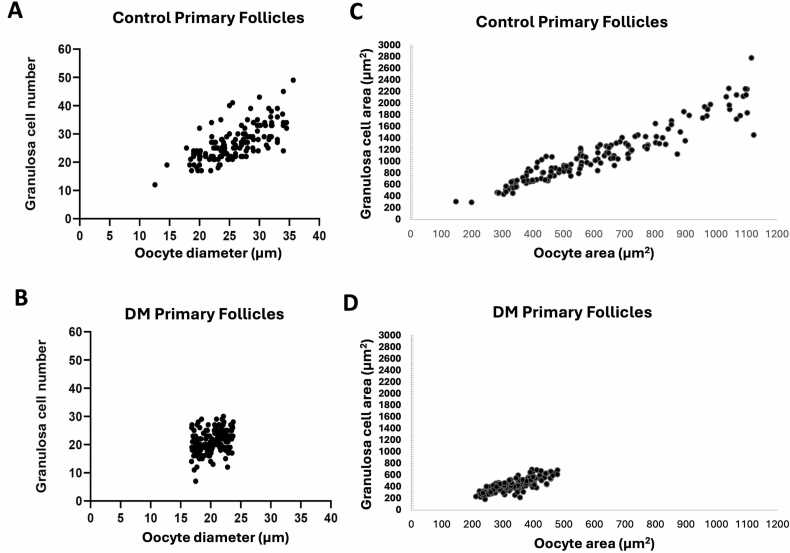
Correlation of Granulosa cell number and oocyte diameter of primary follicles between control (A) and DM ovaries (B) and Granulosa cell area and oocyte area of primary follicles between control (C) and DM ovaries (D). Follicles were derived from n = 3 ovarian grafts per group.

To investigate the relationship between oocyte growth and GC proliferation, we analysed the correlation between oocyte diameter and GC number in primary follicles—the stage at which there is a clear phenotypic effect (Fig. 3). In control primary follicles (n = 148), there was a strong positive correlation between oocyte diameter and GC number (r = 0.69; Fig. 3A), indicating coordinated growth between the oocyte and surrounding somatic cells. In contrast, DM primary follicles (n = 166) also showed a positive correlation, but this was markedly weaker (r = 0.36; Fig. 3B), suggesting impaired coordination between oocyte growth and GC proliferation in the mutant context.

We further investigated the relationship between oocyte and GC areas in primary follicles (Figs. 3C and 3D). A very strong correlation was observed in control follicles (r = 0.95), while DM follicles exhibited a reduced, though still positive, correlation (r = 0.77). Although primordial and transitional follicles also exhibited correlations between oocyte and GC parameters, these were similar between control and DM groups (data not shown).

### 3.3 Evaluation of Granulosa Cells Using AMH and FOXL2 Markers

**Fig. 4:**
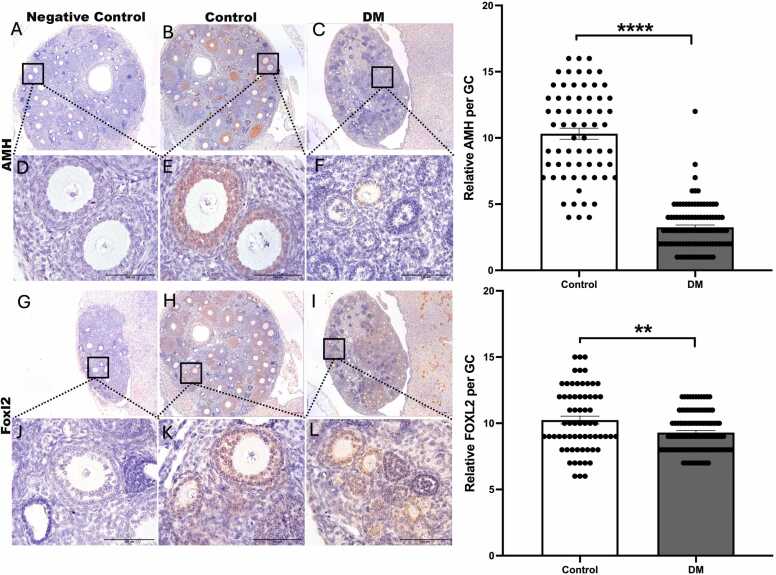
Detection of AMH and FOXL2 in control and DM ovaries. Representative immunohistochemistry images showing the AMH and FOXL2 in ovarian tissue from control and DM mice. For AMH staining, panels A (4X) and D (40X) show the negative control; panels B (4X) and E (40X) show AMH detected in control ovaries; and panels C (4X) and F (40X) show AMH detected in DM ovaries. For FOXL2 staining, panels G (4X) and J (40X) show the negative control; panels H (4X) and K (40X) show FOXL2 detected in control ovaries; and panels I (4X) and L (40X) show FOXL2 detected in DM ovaries. Follicles were derived from n = 3 ovarian grafts per group. Graphs present relative AMH and relative FOXL2 per Granulosa Cell (GC). Results are presented as mean ± SEM. **p < 0.01, ****p < 0.0001.

AMH is a marker of GC functionality and is produced by GCs of growing follicles, primarily at the preantral and small antral stages. Therefore, AMH was used to evaluate the extent of follicular development and the presence of functional GCs in developing follicles in both control and DM groups. Immunohistochemical analysis revealed strong AMH staining in control GCs whereas DM follicles exhibited a reduced level (p < 0.0001) (Figs. 4A, 4B, 4C, 4D, 4E, and 4F). The lower AMH in DM follicles compared to controls suggests compromised GC function and impaired progression of follicular development in the mutant group.

FOXL2 is a marker of early GC development and differentiation. Its expression is critical for primordial follicle activation and subsequent stages of follicle development. Therefore, FOXL2 was used to assess the presence and differentiation of GCs, particularly in early-stage follicles, across control and DM groups. FOXL2 expression was decreased in DM GCs compared to controls (p < 0.005; Figs. 4G, 4H, 4I, 4K, 4K and 4L). These suggests that the early stages of follicle development are also significantly affected by the mutation.

## 4 Discussion

In this study, autoimmunity was investigated as a possible cause of POI onset, in the DM mouse model. Since POI results from oocyte-specific gene deletion, if autoimmunity is causing the onset of POI, this would have to be due to some produced or secreted molecules from the oocytes or growing follicles. Thus, to assess the role of the immune system in DM mice, ovaries were removed from neonatal pups on the day of birth and transplanted to an immunocompromised host to determine if removal of a functional immune system resulted in normal follicle development in DM female mice. To test this hypothesis, we assessed follicle development 3 months post-transplantation.

Although no follicles were present at later stages in DM ovaries, in-depth analysis of the early stages revealed some interesting differences in early follicle development with smaller oocytes and fewer GCs around follicles at the same morphological stage of development. These findings show that the mutation compromises the tightly coupled growth dynamics between oocyte growth and GC-proliferation at the primary follicle stage. In control follicles, oocyte enlargement is closely matched by an increase in GC number and area, reflecting coordinated development. However, in DM follicles, this coordination is weakened, suggesting that the mutation interferes with the signaling or mechanisms that normally ensure synchronous growth between the oocyte and its surrounding somatic cells.

Immunohistochemical analyses revealed that both FOXL2 and AMH expression were significantly reduced in DM ovaries compared to controls, indicating impaired granulosa cell differentiation and disrupted follicle maturation. While initial GC formation may occur, the reduction in these key markers reflects compromised oocyte–GC signaling and altered follicular development. These findings demonstrate that the POI phenotype in DM mice arises from intrinsic defects within the oocyte–follicle unit rather than immune-mediated mechanisms, highlighting the critical role of oocyte-derived cues in regulating granulosa cell function and follicle progression.

Interestingly, although oocytes were smaller in size in transplanted DM ovaries compared to controls whereas when grown in situ in DM mice, at 3 months, there was no difference in oocyte size (Sheikh et al., 2023), both AMH and FOXL2 expression were significantly reduced in DM follicle. This suggests that disrupted oocyte–granulosa cell signaling contributes to impaired follicle growth and maturation. It remains intriguing to speculate on the mechanisms driving these changes, but nevertheless, the POI phenotype persists in DM ovaries even in an immunocompromised environment, underscoring the intrinsic nature of the defect.

Therefore, in the absence of a functional immune system, DM ovaries still exhibited impaired follicle development compared to controls clearly indicating that the cause of POI in DM females is not due to autoimmunity. These findings confirm that the oocyte-specific mutation in DM mice exerts a direct, intrinsic effect on follicle development, independent of immune system involvement (Ackert et al., 2001, Commin et al., 2012, Wei et al., 2024).

Our findings extend previous work on the DM model, in which oocyte-specific deletion of *Mgat1* and *C1galt1* disrupts follicle development and leads to early infertility. Prior studies have shown that DM ovaries contain more primary follicles with impaired granulosa cell proliferation and that these follicles fail to progress, likely due to disrupted oocyte–somatic cell communication (Sheikh et al., 2023). Transcriptomic analyses further support the role of altered cell–cell communication and extracellular matrix pathways in driving ovarian dysfunction (Kaune et al., 2022). Transplantation of ovarian tissue from 9-week-old control and DM mice to immunocompatible sibling control females did not restore follicle development, however, if DM POI is due to autoimmunity, we need to transplant the ovaries before they have been exposed hence the current study. Thus, by analysis of neonatal DM ovaries transplanted into SCID hosts for 3 months, we demonstrate that follicle development remains deficient despite the absence of functional T and B lymphocytes (Bosma et al., 1983, Ackert et al., 2001, Commin et al., 2012, Wei et al., 2024). These data indicate that the ovarian phenotype in DM mice is not initiated by autoimmune attack but instead reflects intrinsic defects within the oocyte–follicle unit. Although the persistence of follicle growth arrest in SCID recipients indicates a novel conceptual advance that adaptive immune cells are not required for the observed phenotype, this approach cannot definitively exclude more subtle immune-mediated effects or contributions from innate immunity. Future studies incorporating targeted analysis of immune cell infiltration will be important to address this limitation.

Kidney capsule transplantation into SCID hosts provides a robust approach to examine ovarian function in the absence of adaptive immunity. This model offers several advantages, including a well-vascularized site for graft survival enabling transplanted tissues to develop *in vivo* and notably in this study, exposure of both DM and control tissues to the same host environment controlling for systemic effects. However, we acknowledge there are some limitations: SCID mice may display subtle endocrine differences independent of immune function, the transplantation procedure may introduce local stress or ischemia, and innate immune components remain functional, so minor immune contributions cannot be completely excluded. Despite these caveats, the persistent block in follicle development observed in DM grafts, but not controls, indicates that the phenotype primarily reflects intrinsic oocyte/follicle defects. Combining this approach with targeted immune cell characterization in future studies will further strengthen the link between structural abnormalities and functional ovarian insufficiency.

Clinically, POI is defined primarily by functional criteria, including menstrual disturbance and biochemical evidence of hypothalamic–pituitary–ovarian axis disruption, most commonly elevated FSH, and reduced AMH and estradiol concentrations. The progressive decline in follicle development accompanied by changes in endocrine profiles have established the DM mouse as a model of POI (Williams and Stanley, 2011). In the present study, the focus was on analyses of the ovarian tissue in an environment lacking adaptive, but not innate, immunity since it was the ability of follicles to develop that we were assessing. Thus, in order to provide the optimum environment for follicle development, the host mice were left intact – i.e., not ovariectomized. Therefore, in this model, analyses of endocrine profiles were not carried out in the transplanted cohort, and we used detailed histological and quantitative readouts to determine ovarian competence. The marked depletion of follicles, increased atresia, and reduced follicle and oocyte size collectively indicate impaired follicle growth and survival, representing structural correlates of ovarian insufficiency. It is possible that SCID mice may exhibit alterations in endocrine physiology independent of immune function, which could potentially influence graft outcomes – however, we are unaware of such reports since the mice exhibit normal fertility as these mice are viable and maintained as breeding colonies with documented pregnancy and reproductive performance by supplier Envigo (https://www.inotiv.com/research-model/cb-17-icrhan-hsd-prkdcscid). Nevertheless, because both control and DM ovaries were transplanted into the same recipient background, any systemic SCID-related effects are expected to influence both groups similarly and follicles in the control ovaries developed normally. Although these morphological findings cannot fully substitute for endocrine confirmation, they are consistent with features reported in ovaries from patients with POI and provide mechanistic insight into graft dysfunction.

## 5 Conclusion

In conclusion, our study demonstrates that the onset of POI in the DM mouse model is not driven by autoimmunity but rather results from intrinsic defects in oocyte function. Specifically, oocytes lacking the ability to generate core 1 O-glycans and complex/hybrid N-glycans fail to properly support granulosa cell function, leading to impaired follicle growth and maturation. These findings highlight the critical role of oocyte–granulosa cell interactions in maintaining ovarian function and provide mechanistic insight into the pathogenesis of POI.

## CRediT authorship contribution statement

**Williams Suzannah Alice:** Writing – review & editing, Visualization, Validation, Supervision, Resources, Project administration, Methodology, Investigation, Funding acquisition, Data curation, Conceptualization. **Sergej Petrovic:** Writing – review & editing, Visualization, Validation, Project administration, Methodology, Investigation, Formal analysis, Data curation. **Nilay Kuscu:** Writing – review & editing, Writing – original draft, Visualization, Validation, Supervision, Resources, Project administration, Methodology, Investigation, Formal analysis, Data curation, Conceptualization. **Ruth Appeltant:** Writing – review & editing, Methodology, Conceptualization.

## Funding

This work was supported by grants from the 10.13039/501100000265Medical Research Council to S.A.W (G0900058 and G0900058/1) and by grant number 2021–238038 from the Chan Zuckerberg Initiative DAF, an advised fund of 10.13039/100000923Silicon Valley Community Foundation, to fund N. K. This work also was funded by Fondation Hoffmann to S.A.W. supporting R.A., the Covid-19 rebuilding research momentum fund awarded to R.A. (project reference 0009963) and Nuffield Department of Women’s and Reproductive Health funding to S.A.W.

## Declaration of Competing Interest

Suzannah Williams reports financial support was provided by Medical Research Council. Suzannah Williams reports financial support was provided by Fondation Hoffman. Suzannah Williams reports financial support was provided by The Chan Zuckerberg Initiative. Ruth Appeltant reports financial support was provided by Covid-19 rebuilding research momentum fund. Reports a relationship with that includes:. Has patent pending to. If there are other authors, they declare that they have no known competing financial interests or personal relationships that could have appeared to influence the work reported in this paper.

## References

[bib1] Ackert C.L., Gittens J.E., O’Brien M.J., Eppig J.J., Kidder G.M. (2001). Intercellular communication via connexin43 gap junctions is required for ovarian folliculogenesis in the mouse. Dev. Biol..

[bib2] Adeniran B.V., Bjarkadottir B.D., Appeltant R., Lane S., Williams S.A. (2021). Improved preservation of ovarian tissue morphology that is compatible with antigen detection using a fixative mixture of formalin and acetic acid. Hum. Reprod..

[bib3] Bosma G.C., Custer R.P., Bosma M.J. (1983). A severe combined immunodeficiency mutation in the mouse. Nature.

[bib4] Buigues A., Marchante M., Herraiz S., Pellicer A. (2019). Diminished ovarian reserve chemotherapy-induced mouse model: A tool for the preclinical assessment of new therapies for ovarian damage. Reprod. Sci..

[bib5] Chapman C., Cree L., Shelling A.N. (2015). The genetics of premature ovarian failure: current perspectives. Int. J. Women’S. Health 7.

[bib6] Commin L., Buff S., Rosset E., Galet C., Allard A., Bruyère P., Joly T., Guérin P., Neto V. (2012). Follicle development in cryopreserved bitch ovarian tissue grafted to immunodeficient mouse. Reprod. Fertil. Dev..

[bib7] Crowe A.R., Yue W. (2023). Semi-quantitative determination of protein expression using immunohistochemistry staining and analysis. Bioprotoc..

[bib8] Durlinger A.L., Visser J.A., Themmen A.P. (2002). Regulation of ovarian function: the role of anti-Müllerian hormone. Reproduction.

[bib9] Grasa P., Kaune H., Williams S.A. (2012). Embryos generated from oocytes lacking complex N- and O-glycans have compromised development and implantation. Reproduction.

[bib10] Grasa P., Ploutarchou P., Williams S.A. (2015). Oocytes lacking O-glycans alter follicle development and increase fertility by increasing follicle FSH sensitivity, decreasing apoptosis, and modifying GDF9:BMP15 expression. FASEB J..

[bib11] Grasa P., Sheikh S., Krzys N., Millar K., Janjua S., Nawaggi P., Williams S.A. (2016). Dysregulation of follicle development in a mouse model of premature ovarian insufficiency. Reproduction.

[bib12] Grossmann B. (2020). Immunological aspects of premature ovarian insufficiency. Endocr. Rev..

[bib13] Hoek A., Schoemaker J., Drexhage H.A. (1997). Premature ovarian failure and ovarian autoimmunity. Hum. Reprod. Update.

[bib14] Hsieh Y.T., Ho J.Y.P. (2021). Thyroid autoimmunity is associated with higher risk of premature ovarian insufficiency-a nationwide Health Insurance Research Database study. Hum. Reprod..

[bib15] Hubayter Z., Popat V.B., Nelson L.M. (2010). Hypoestrogenism and the brain: what can we learn from a rodent model of menopause?. Front. Biosci. (Elite Ed. ).

[bib16] Ju T., Cummings R.D., Canfield W.M. (2002). Purification, characterization, and subunit structure of rat core 1 Beta1,3-galactosyltransferase. J. Biol. Chem..

[bib17] Kaune H., Montiel J.F., Fenwick M., Williams S.A. (2022). Rapid ovarian transcript changes during the onset of premature ovarian insufficiency. Reprod. & Fertil..

[bib18] Li H., You L., Tian Y., Guo J., Fang X., Zhou C., Shi L., Su Y.Q. (2020). DPAGT1-Mediated protein N-Glycosylation is indispensable for oocyte and follicle development in mice. Adv. Sci..

[bib19] Lo B., Yu J., Lu Z. (2019). Glycans in the mammalian oocyte and preimplantation embryo. Biol. Reprod..

[bib20] Lv S.J., Hou S.H., Gan L., Sun J. (2021). Establishment and mechanism study of a primary ovarian insufficiency mouse model using lipopolysaccharide. Anal. Cell. Pathol..

[bib21] Meskhi A., Seif M.W. (2006). Premature ovarian failure. Curr. Opin. Obstet. Gynecol..

[bib22] Nelson L.M. (2009). Clinical practice. Primary ovarian insufficiency. N. Engl. J. Med..

[bib23] Nelson L.M., Anasti J.N., Kimzey L.M. (1994). Development of luteinized Graafian follicles in patients with karyotypically normal spontaneous premature ovarian failure. J. Clin. Endocrinol. & Metab..

[bib24] Rudnicka E., Kruszewska J., Klicka K. (2018). Premature ovarian insufficiency–aetiopathology, epidemiology, and diagnostic evaluation. Prz. Menopauzalny.

[bib25] Schmidt D., Ovitt C.E., Anlag K. (2004). The murine winged-helix transcription factor Foxl2 is required for granulosa cell differentiation and ovary maintenance. Development.

[bib26] Setiady Y.Y., Samy E.T., Tung K.S. (2003). Maternal autoantibody triggers de novo T cell-mediated neonatal autoimmune disease. J. Immunol..

[bib27] Sharif K., Watad A., Bridgewood C., Kanduc D., Amital H., Shoenfeld Y. (2019). Insights into the autoimmune aspect of premature ovarian insufficiency. Best practice & research. Clin. Endocrinol. & Metab..

[bib28] Sheikh S., Lo K.B., Kaune H., Deleva J., Williams S. (2023). Rescue of follicle development after oocyte induced ovary dysfunction and infertility in a model of POI. Front. Cell Dev. Biol..

[bib29] Shi S., Williams S.A., Seppo A., Kurniawan H., Chen W., Ye Z., Marth J.D., Stanley P. (2004). Inactivation of the *Mgat*1 gene in oocytes impairs oogenesis, but embryos lacking complex and hybrid N-glycans develop and implant. Mol. Cell. Biol..

[bib30] Shuster L.T., Rhodes D.J., Gostout B.S. (2010). Premature menopause or early menopause: Long-term health consequences. Maturitas.

[bib31] Suzuki N., Yoshioka N., Takae S. (2015). Preservation of the ovarian reserve in cancer patients. Reproduction.

[bib32] Tung K.S.K., Teuscher C., Meng A.L. (1997). Autoimmune oophoritis, ovarian antigen, and diseases of autoimmune origin. Am. J. Reprod. Immunol..

[bib33] Vujovic S. (2009). Aetiology of premature ovarian failure. Menopause Int..

[bib34] Wang X.F., Zhang L., Wu Q.H., Min J.X., Ma N., Luo L.C. (2015). Biological mechanisms of premature ovarian failure caused by psychological stress based on support vector regression. Int. J. Clin. Exp. Med..

[bib35] Wassarman P.M., Litscher E.S. (2021). Zona pellucida genes and proteins: Essential players in mammalian oogenesis and fertility. Genes.

[bib36] Weenen C., Laven J.S., Von Bergh A.R. (2004). Anti-Müllerian hormone expression pattern in the human ovary: potential implications for initial and cyclic follicle recruitment. Mol. Hum. Reprod..

[bib37] Wei X., Bjarkadottir B.D., Nadjaja D., Sheikh S., Fatum M., Lane S., Williams S.A. (2024). Effect of AMH on primordial follicle populations in mouse ovaries and human pre-pubertal ovarian xenografts during doxorubicin treatment. Front. Cell Dev. Biol..

[bib38] Williams S.A., Stanley P. (2009). Oocyte‑specific deletion of complex and hybrid N‑glycans leads to defects in preovulatory follicle and cumulus mass development. Reproduction.

[bib39] Williams S.A., Stanley P. (2011). Premature ovarian failure in mice with oocytes lacking core 1-derived O-glycans and complex N-glycans. Endocrinology.

[bib40] Williams S.A., Xia L., Cummings R.D., McEver R.P., Stanley P. (2007). Fertilization in mouse does not require terminal galactose or N-acetylglucosamine on the zona pellucida glycans. J. Cell Sci..

[bib41] Zhang C., Yu D., Mei Y., Liu S., Shao H., Sun Q., Lu Q., Hu J., Gu H. (2023). Single-cell RNA sequencing of peripheral blood reveals immune cell dysfunction in premature ovarian insufficiency. Front. Endocrinol..

